# Smart pumps improve medication safety but increase alert burden in neonatal care

**DOI:** 10.1186/s12911-019-0945-2

**Published:** 2019-11-07

**Authors:** Kristin R. Melton, Kristen Timmons, Kathleen E. Walsh, Jareen K. Meinzen-Derr, Eric Kirkendall

**Affiliations:** 1 Division of Neonatology and Pulmonary Biology, Cincinnati Children’s Hospital Medical Center, Cincinnati, OH 45229 USA; 2 Department of Pediatrics, College of Medicine, University of Cincinnati, Cincinnati, OH USA; 3 Division of Hospital Medicine, Cincinnati Children’s Hospital Medical Center, Cincinnati, OH USA; 4 James M. Anderson Center for Health Systems Excellence, Cincinnati Children’s Hospital Medical Center, Cincinnati, OH USA; 5 Division of Biostatistics and Epidemiology, Cincinnati Children’s Hospital Medical Center, Cincinnati, OH USA; 6 Division of Biomedical Informatics, Cincinnati Children’s Hospital Medical Center, Cincinnati, OH USA; 7 Department of Pediatrics, Wake Forest School of Medicine, Winston-Salem, NC USA

**Keywords:** Patient safety, Medication administration errors, Smart infusion pumps, Infusion pump alerts, Alert fatigue

## Abstract

**Background:**

Smart pumps have been widely adopted but there is limited evidence to understand and support their use in pediatric populations. Our objective was to assess whether smart pumps are effective at reducing medication errors in the neonatal population and determine whether they are a source of alert burden and alert fatigue in an intensive care environment.

**Methods:**

Using smart pump records, over 370,000 infusion starts for continuously infused medications used in neonates and infants hospitalized in a level IV NICU from 2014 to 2016 were evaluated. Attempts to exceed preset soft and hard maximum limits, percent variance from those limits, and pump alert frequency, patterns and salience were evaluated.

**Results:**

Smart pumps prevented 160 attempts to exceed the hard maximum limit for doses that were as high as 7–29 times the maximum dose and resulted in the reprogramming or cancellation of 2093 infusions after soft maximum alerts. While the overall alert burden from smart pumps for continuous infusions was not high, alerts clustered around specific patients and medications, and a small portion (17%) of infusions generated the majority of alerts. Soft maximum alerts were often overridden (79%), consistent with low alert salience.

**Conclusions:**

Smart pumps have the ability to improve neonatal medication safety when compliance with dose error reducing software is high. Numerous attempts to administer high doses were intercepted by dosing alerts. Clustered alerts may generate a high alert burden and limit safety benefit by desensitizing providers to alerts. Future efforts should address ways to improve alert salience.

## Background

Medication errors are common in pediatrics, and errors with the potential to cause harm are significantly increased in neonates cared for in intensive care environments [1]. Younger and critically ill patients are also more likely to suffer harm from a medication error [1, 2]. Neonates are a particularly vulnerable population, due to many factors, including their physiologic immaturity and rapidly changing weights that affect weight-based medication dosing [1, 3]. For this reason and others, hospitals have quickly and widely adopted technologies that have the potential to reduce medication errors in the neonatal intensive care unit (NICU).

Smart pumps are a promising technology to prevent medication administration errors. Smart pumps are infusion pumps manufactured with software that checks the nurse-programmed medication administration against pre-established institutional limits in customized medication libraries before beginning infusion. Because administration is the last step in the medication use pathway after ordering and dispensing, there are few opportunities to intercept errors in administration. As such, nurse administration errors are more likely to reach the patient than physician ordering or pharmacy dispensing errors [4]. Smart pumps provide alerts around many types of potentially unsafe infusion conditions, providing both ‘soft alerts’ and ‘hard stops’ when programmed dosing limits are violated. The goal of soft alerts is to raise awareness by notifying the user that the programmed medication infusion parameters are outside of the usual dosing range. These alerts can be overridden, however, and the infusion continued. Hard stops notify the user that the medication infusion parameters are outside of safe limits and force cancellation of the infusion. The American Society of Health-System Pharmacists national survey of 2014 suggested that 80.5% of surveyed hospitals in the US use smart pumps, up from 32.2% in 2005, demonstrating the rise in use of smart pumps [5, 6]. Despite rising rates of adoption, little data exists on the impact of smart pump alerts on medication safety and alert fatigue in the pediatric and neonatal populations.

Some studies have raised the concern that smart pumps may actually introduce error themselves and require a high rate of compliance to observe safety benefits [7–9]. As with all technology, the implementation of smart pumps may come with unintended consequences, and organizational structure, the technology itself or patient needs may drive the development of workarounds [10, 11]. Issues that have been raised in the literature include workarounds like the use of basic infusions that do not employ the drug library dosing limits, improper patient or medication identification during pump programming, and high override rates for soft alerts, all of which negate the safety features of smart pumps [8, 12]. The use of an additional alerting system in the ICU environment also raises the concern of alert fatigue and the salience of smart pump alerts, due to suboptimal levels of actionable alerts [13]. As a result of alert fatigue from cognitive overload or densitization with time, responsiveness to smart pump alerts may be influenced by other factors, such as work shift, month, repetitiveness of alerts or work complexity [14, 15]. The goal of this study was to assess whether smart pumps prevent medication errors for continuous intravenous medications used in the neonatal population and to assess whether smart pumps are a source of alert burden in the neonatal intensive care unit (NICU). The immediate goal of this study was to characterize the data that smart pumps generate, describe how it changes over time, and understand whether contextual factors, such as work shift, influence the data. We chose to focus our work on neonates as one of the most vulnerable populations but anticipate broader pediatric applicability.

## Methods

### Study site and patient population

The study took place in a level four NICU that provides the highest level of neonatal intensive care to complex and critically ill newborns requiring extracorporeal mechanical oxygenation (ECMO), surgical and subspecialty care. At the time of the study, the NICU had 59 registered beds and an average of 725 yearly admissions. Patients had an average gestational age of 35 weeks and an average length of stay of 23 days.

NICU safety technologies in place at the time of the study include the use of computerized provider order entry with electronic medication administration records and clinical decision support, a barcode medication administration system, smart infusion pump technology with a customized neonatal library of 158 medications, daily rounding and prescription review by dedicated NICU pharmacists and clinical guidelines for high-risk medications.

### Data sources

Alaris™ (Becton, Dickinson, and Company, Franklin Lakes, NJ) intravenous infusion smart pumps with dose error reduction software (DERS) were introduced in January of 2014. Data from pumps was wirelessly downloaded daily and distributed for analysis monthly. Pump reports included the pump identification number (Point-of-Care Unit and module), infusion number, patient medical record number, drug name, drug concentration and rate, alerts, alarms and actions, and date and time stamps for all actions. Each action or change the infusion pump undergoes is captured in a smart pump record (SPR). The NICU medication library contained in the smart pumps was customized in 2013 through a collaborative effort with neonatologists, pharmacists, and neonatal nurse practitioners. Smart pump libraries and medication issues were reviewed monthly by a multidisciplinary medication use team to assess medication additions and adequacy of dosing limits. Changes to the library have been tracked since 2014 with updates occurring on average 4–5 times/year. Sixty percent of medications and fluids in the library have hard maximum limits, and this has been consistent over time.

### Study design/methodological approach

We evaluated retrospective smart pump data for all neonatal patients from January 2014–December 2016, which included 727, 745 and 655 patients by year. We focused our evaluation on continuous medications since these medications typically represent high-risk medications and have been the emphasis of our previous area of study. The data presented are limited to smart pump data on continuous medications and their associated medication alerts. We sought to understand the baseline data generated by smart pumps in the NICU and to understand how dynamic the data were over the study period through use of descriptive statistics and trend analysis. Patterns in the data were examined to determine the dynamic nature of the system and the reliability of the system, the quality of the data, alert burden, response to alerts, and contextual effects, such as shift or day of the week, on the data.

## Measures

Data measures evaluated were as follows:

- **Infusion Frequency**: number of unique infusions and number of infusions started
- **Alert Frequency**: number of alerts and percentage of alerts per infusions started
- **Compliance with Drug Library**: percent compliance of clinician programming the pump with drug library utilization
- **User Response to Pump Alerts:** actions by percentage and number
- **Alert Burden by Time**: alert number and percentage by shift, day, week/weekend, and month.
- **Alert Burden by Medication**: alert number and percentage by medication
- **Salience of Alerts**: We calculated alert salience as one measure of the effectiveness of alerts as it measures the extent to which a provider takes corrective action in response to the presented alert. Alert salience has been used to demonstrate provider response to dosing alerts issued during medication ordering and is suggested to be a proxy for alert fatigue [16, 17]. We calculated alert salience as the number of cancelled or reprogrammed infusions/total number of alerts. A low alert salience indicates that a high number of alerts are being generated but do not result in significant action.
- **Alert Burden by Library Threshold Violation:** number of ‘soft maximum’ and ‘hard maximum’ violations by year and user response by type of violation, as well as number of ‘soft minimum’ and ‘hard minimum’ violations.
- **Percent variance from soft and hard maximum thresholds**: percent variance over soft and hard maximum thresholds

## Analysis

Alerts were analyzed in two ways: 1) the frequency and percentage of infusion starts that had an associated alert (total number of alerts/total number of infusion starts) and 2) the frequency and percentage of unique infusions that had at least one associated alert (at least one alert/number of unique infusions) (Table 1). Data distributions regarding the frequency and percentage of alerts were reported by year. Potential temporal changes in the percentages of alerts across years were tested with chi-square test of trend. The response to the alerts were classified as either canceled, overridden, or reprogrammed (Table 1) and described across the three study years. The percent of infusions with alerts was also assessed by the nursing shift, day of the week, and weekday versus weekend. Alert burden by medication was also evaluated by reviewing the distributions of alerts across the various medications.

**Table 1:** Definitions

Unique and Total Infusion Starts	Each new bag or syringe for a medication is given a unique infusion ID. Given that a unique infusion may be started and stopped multiple times, as a result of alarms and alerts, the total number of infusion starts will be greater than the number of unique infusions. We considered each infusion start an opportunity to evaluate the pump.
Cancel	Provider cancels the pump programming after an alert and starts over. Cancellation is forced by the pump when a hard limit is exceeded.
Reprogram	Provider resets the pump programming for medication dose or rate after an alert.
Override	Provider receives an alert and chooses to proceed without changes in pump programming.
Basic Mode	Pump programming mode that requires all information to be manually entered and which bypasses all safety features of the pump except volume to be infused.
Dose Error Reduction Software	Software present in the pump that allows the creation of a population-specific drug library with established minimum and maximum dose and/or rate limits by medication.

## Results

### System stability and drug library compliance

We had a consistent proportion of unique infusions and infusion starts per year over the period of 2014–2016 (Table 2). The decrease in numbers in 2015 is attributed to a data transmission issue from August to December of 2015, during a system update that led to lower than usual smart pump record numbers. Compliance with the drug library was high, with an average of 87% of medications (84–89%) infused using the library program with dose error reduction software (Table 2). Infusions were run in the basic mode, lacking DERS safety features, an average of 13% (11–16%) of the time.

**Table 2:** Infusion and Alert Data

Variable	2014	2015	2016	All Years Average
# of Infusion Starts	137,423	85,006^+^	147,876	123,435
# of unique infusion IDs	103,284	57,932^+^	103,254	88,157
# of Infusion Starts Using DERS	89%	84%	87%	87%
# of Infusion Starts Using Basic Mode	11%	16%	13%	13%
% Infusion Starts with Alerts	2.80%	5.40%*	4.90%*	4.37%
Response to Alerts				
Override	71.40%	71.10%	76.40%*	73.6%
Cancel	23.80%	25.70%	21.50%*	23.3%
Reprogram	4.80%	3.10%	2.20%*	3.1%
% of Alerts by Shift				
Day	32.00%	30.40%	33.10%	32.10%
Evening	40.20%	38.30%	40.30%	39.70%*
Night	27.90%	31.30%	26.60%	28.30%
% of Infusion Starts with Alerts by shift				
Day	2.90%	5.40%	5.10%	4.30%
Evening	2.90%	4.90%	4.90%	4.20%
Night	2.80%	6.00%	4.70%	4.30%

(+) identifies data transmission loss in 2015, (*) *p* < 0.0001

### Alerts, response to alerts and overall alert burden

Alerts occurred in an average of 4.3% of all infusion starts (range 2.8–5.4% across the three years) with 15,720 alerts for 264,470 unique infusions. There was a small but statistically significant change in the percentage of infusion starts with alerts that occurred each year (*p* < 0.0001; Table 1). An average of 2.5% of unique infusions had alerts (range 2.0–2.9% across three years, *p* < 0.0001).

The majority of alerts were overridden with an average of 73.6% of alerts overridden (range 71.1–76.4%), as shown in Table 2. An average of 23.3% (21.5–25.7%) of alerts were cancelled and 3.1% (2.2–4.8%) reprogrammed. The responses to alerts showed a small statistical change over time by year, with more alerts being overridden and fewer being cancelled or reprogrammed in 2016 when compared to 2014 (*p* < 0.0001).

To understand the effect of context on alerts, we evaluated the effect of work shift, day and month. There was a statistically significant (*p* < .0001) difference regarding the overall number and percent of alerts by shift across the three years, with a higher proportion of alerts occurring on evening shift. However, the percent of infusion starts with alerts did not differ by shift. (*P* = 0.17; Table 2) The percentages of infusions with alerts also did not vary significantly by day of the week, or weekday versus weekend (*p* = 0.38; not shown).

**Fig. 1:**
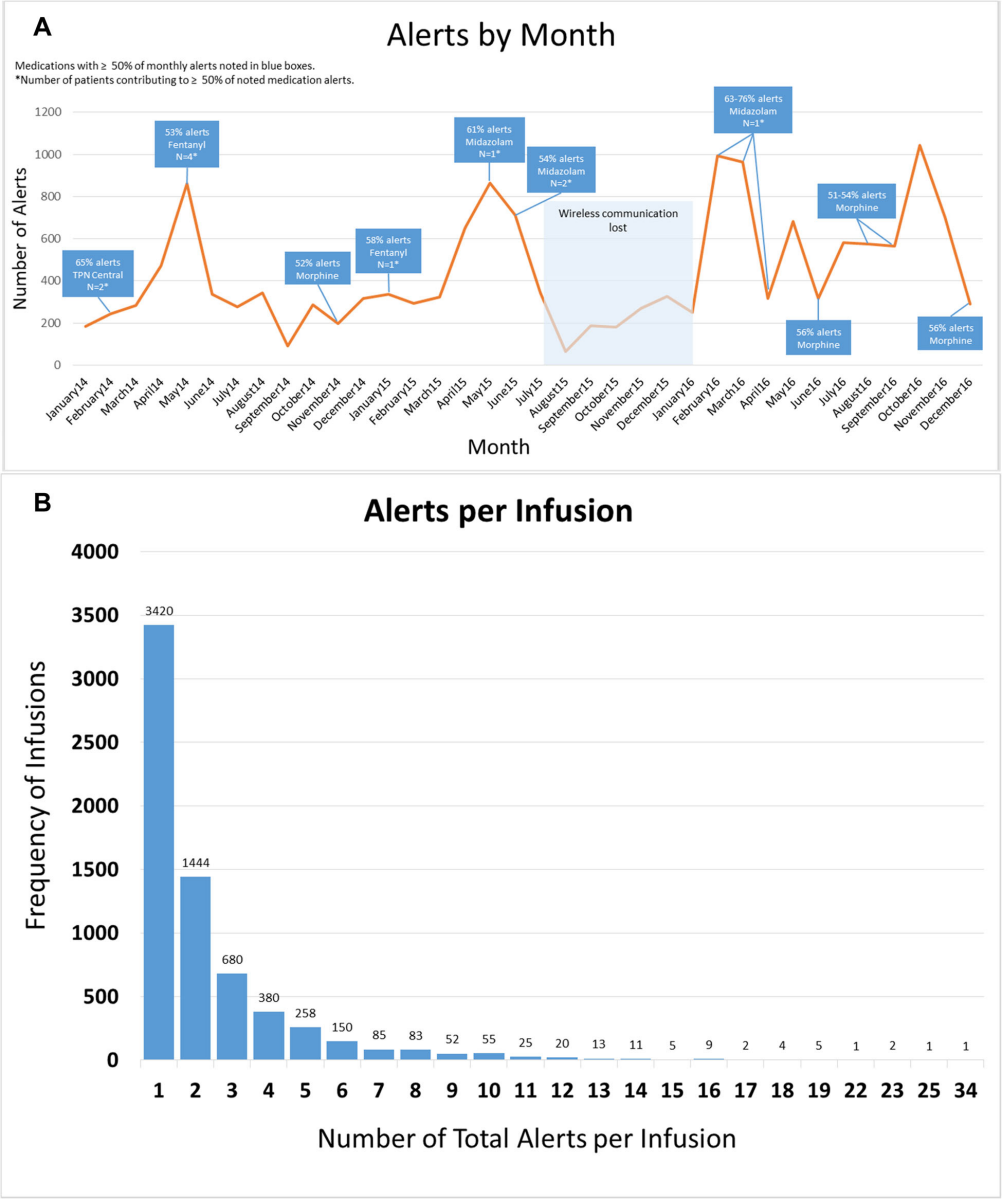
**a** Alert Frequency by Month with Clustered Medication and Patient Alerts. Trends of total alerts by month are shown, highlighting medications that contributed to over 50% of the alerts in that month. When less than 5 patients contributed over 50% of alerts for a high-alerting medication, the number of patients is identified. **b** – Number of Alerts per Infusion. The graph demonstrates the number of infusions that generated a single alert versus multiple alerts. Most infusions had a single associated alert, but 17% of infusions generated multiple alerts (4–34 alerts) per infusion

However, when analyzing the pump alerts by month, significant variation occurred (Fig. 1a). We evaluated individual months that had high alert burdens and found that a single patient, and even a single medication for that patient, could generate a very high alert burden. In one example, one patient accounted for 75% of all alerts from one month with 412 of 523 midazolam alerts and 415 of 415 hydromorphone alerts (Fig. 1a).

**Fig. 2:**
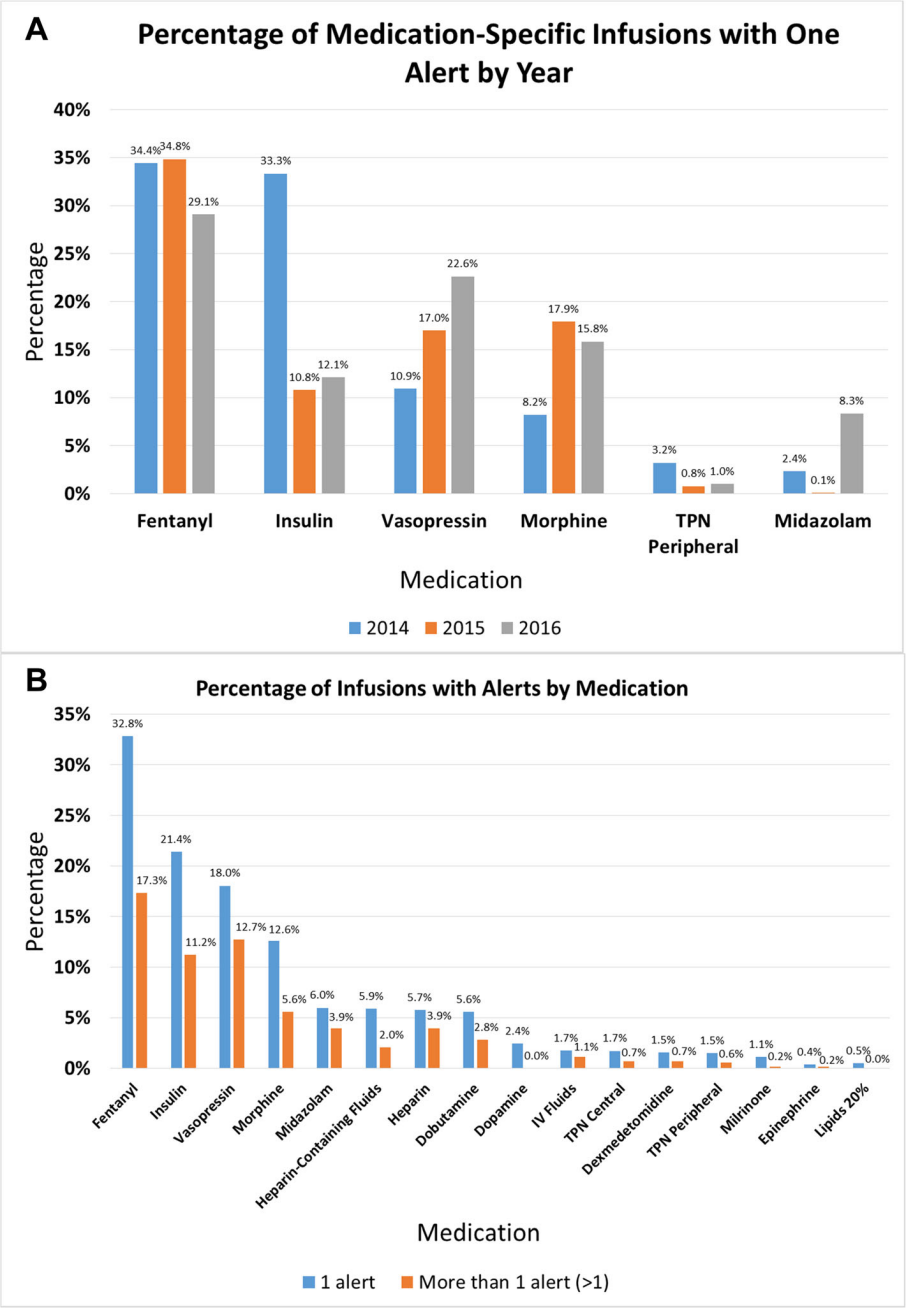
**a** Top Alerting Medications. The top six medications with alerts per year are shown, along with the percentage of infusion starts generating alerts per year. **b** – Multiple Alerts. The percentage of infusion starts that generated one alert, or multiple alerts, by medication. The medications that generated the highest number of alerts per infusion also had high numbers of infusions with multiple alerts

To evaluate further the issue of clustered high alert burdens, we evaluated the number of alerts that occurred per infusion. We found that 51% of infusions with alerts generated 1 alert, 21.5% had 2 alerts, 10% had 3 alerts and the remaining 17% of infusions with alerts had 4–34 alerts (Fig. 1b). Therefore, a small number of infusions generated a large number of alerts. Fentanyl, vasopressin and insulin were the medications most likely to have > 3 alerts per infusion (Fig. 2b).

### Alert burden by medication

We evaluated medications that generated the highest number of alerts per infusion starts as a reflection of their dosing rules. Fentanyl, insulin, vasopressin, morphine, TPN and midazolam generated the highest number of alerts per infusion starts (Fig. 2a) and demonstrated a large number of infusions with both one alert and multiple alerts (Fig. 2b). There was some variation in the percent of medication infusions that generated alerts by year, but the top 6 medications remained essentially the same during the time period studied (Fig. 2a). Override rates remained high (63.1–84.8%) for medications with the highest alert rates and similar to all medications with an average override rate at 74.7% (data not shown).

### Alert salience

**Fig. 3:**
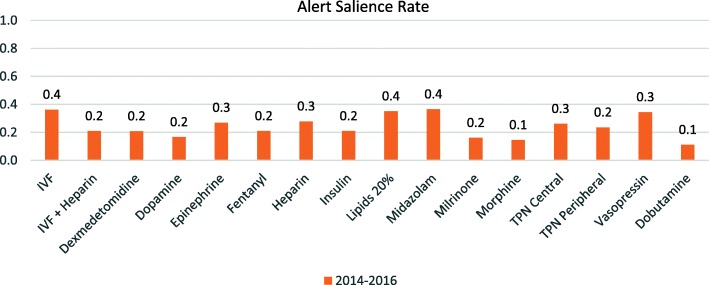
Alert Salience Rates for Individual Medications. Salience rates were calculated for each study medication by determining the number of cancelled or reprogrammed infusions/total number of alerts for an individual medication

As a means to assess the effectiveness of infusion alerts, we assessed alert salience. As shown in Fig. 3, alert salience differed significantly across medications. Some high-risk medications such as fentanyl and morphine exhibited salience rates as low as 10–20%. In general, most salience rates for the studied medications were 30% or less. One limitation to this measure is that cancelled infusions may be restarted as basic infusions that will not generate further alerts, which could skew the results, but our high rate of DERS usage and low rate of basic infusions limits the effect on our results.

### Drug library threshold violations

To assess potentially prevented errors, we evaluated the number of attempts to exceed hard limit maximums. We identified 160 attempts to exceed the hard limit maximum, which is the type of threshold violation that carries the most risk to patients. We performed individual manual review of 20 hard stops and found that 15 (75%) were true discrepancies while 5 (25%) could be contributed to attempts at rapid pump priming (data not shown). We also identified 2093 attempts to exceed the soft limit maximum that resulted in the infusion being reprogrammed or cancelled (Table 3).

**Table 3:** Drug Library Threshold Violations by Type, Year, and User Response

	2014	2015	2016	2014–2016 Total	Cancel	Override	Reprogram
Soft Min	2259	1529	1570	5358	1741	3500	117
Hard Min	23	5	6	34	34	0	0
Soft Max	1556	2973	5639	10,168	1724	8075	369
Hard Max	50	46	64	160	160	0	0
Total	3888	4553	7279	15,720	3659	11,575	486

### Percent variance from soft and hard maximum limits

**Fig. 4:**
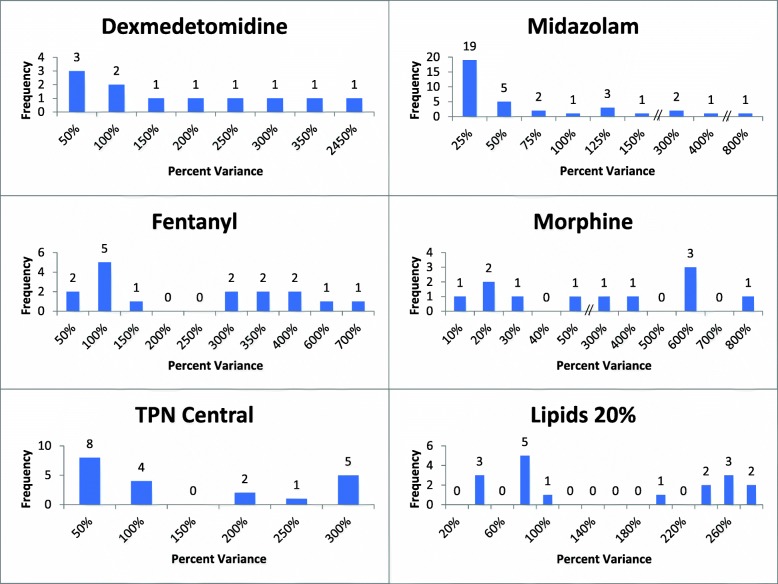
Percent Variance from Soft and Hard Maximum Limits. Sample medication hard maximum threshold violations, by the percent variance. Percent variance represents the magnitude of the attempted infusion dose/rate above the hard maximum threshold of the drug library

We evaluated the doses of programmed infusions that exceeded soft and hard maximum limits to determine the amount by which they exceeded the thresholds. We identified medication infusions that were programmed at doses that varied from the hard maximum dose by as much as 2900% (or 29x the maximum dose), with several high-risk medications such as fentanyl, insulin, morphine, midazolam, and dexmetomidine having administrations programmed at 5-24x the maximum dose. Doses that exceeded the soft maximum dose were highly variable. Many were programmed at doses 20–50% above the soft maximum dose but remained well below the hard maximum limit (Fig. 4).

## Discussion

For neonates, who are at particularly high risk for overdose infusion errors, smart pumps have the potential to be particularly valuable. However, this value is only realized when the pumps and libraries are tailored to use with neonates and the equipment and software are used as intended. Because medication administration errors are more likely to reach the patient, smart pumps have the potential to provide additional protection by double-checking nurse programming. The goal of our study was to assess whether smart pumps are able to prevent medication overdoses in continuous medications administered to the neonatal population. Overall, we found that nurses took advantage of the safety features of smart pumps and programmed the majority of infusions using the drug library with dose error reduction software. This high rate of compliance has been shown to be critical to the effective use of smart pumps [8, 18, 19]. Although our rate of compliance is higher than previously reported rates [12, 13], basic infusions, which lack the safety features of the DERS, were still used for 11–16% of infusions. Targeted discussions with frontline smart pump users revealed that our library had limited options for intravenous fluid selection that did not match the large variety of fluids being used. Work is underway to expand fluid options in our neonatal library, and our experience supports the need for regular reassessment to understand the reasons for basic infusion use in all institutions.

Along with the use of basic infusions, other workarounds that generated alerts were evident in our data. When evaluating hard stops, we found that nurses programmed the pump rate at 2–4 times the dose to facilitate rapid pump priming in 15–30 min instead of one hour. Discussion with the nurses revealed the need for efficiency in the face of urgent patient needs. Smart pumps are not perfect, and workarounds occur when there is a mismatch between the capabilities of a technology and demands of care [20, 21]. Our findings are consistent with the causes of workarounds identified by others, which include patient issues, the need for efficiency, technology-related issues, and organizational issues where policies or libraries do not align with clinical practice [20–22].

Previous studies have used the number of infusions that are reprogrammed or cancelled to assess prevented errors. We found that there were many attempts to exceed the soft maximum limits, less frequent attempts to exceed the hard maximum limits, and that many infusions were cancelled or reprogrammed. Looking specifically at attempts to exceed hard maximums, which we used as a proxy for potentially prevented errors, we found that smart pumps identified and prevented significant overdoses of several high-risk medications, including narcotics, sedatives and insulin, demonstrating their utility in improving patient safety in the NICU. While some hard stops may be contributed to attempts at pump priming, we found examples of incorrect programmed doses, decimal point errors, confusion between two simultaneous medications, and attempts to bolus using the continuous infusion setting. Although these events were infrequent compared to the number of overall infusion starts, the possibility of delivering highly dangerous doses was prevented through the use of the dose error reduction software. This rate of potential error prevention by smart pumps is in line with that reported in other ICU environments [23]. For soft alerts, given that the typical response is to override the alert, cancelling or reprogramming of the infusion is more likely to represent a true prevented error. We found 2093 examples of cancelled or reprogrammed infusions after soft alerts.

The second goal of our study was to assess whether smart pumps are a source of alert burden and potential alert fatigue in the NICU. When looking across the unit as a whole, our data suggest that smart pump alerts for continuous infusions do not contribute significantly to overall alert burden with only 3–5% of infusions alerting per infusion start, which is significantly less than the 12–16 alarms/hour nurses experience from physiologic monitors in the NICU [24, 25]. While there was a statistically significant change in the percentage of alerts per year, it is unlikely that this represents a clinically significant change given the relatively low frequency of infusion-related alerts. When we evaluate alerts from the perspective of the patient, however, we found that alerts cluster around specific patients and specific infusions, with 17% of infusions generating significant alert numbers. In these unique and interesting situations, we found that a single medication in a single patient could repetitively alert, creating a very high alert burden and potentially making it difficult for a caregiver to identify and act upon appropriate alerts representing true errors. The evidence on alert fatigue shows that fatigue is connected to the proportion of repeated alerts [14], potentially creating unique situations where individual patients are placed at increased risk for experiencing missed alerts or delayed responses. In our data, repetitive alerts occurred during the initial start of medications and clustered around patients that required doses of medication that were significantly higher than normal, most commonly at the end of life, when sedatives and analgesics had been significantly escalated. There are patients that will be exceptions to the standards of the library based on their individual physiology or during end-of-life care. Current smart pumps do not have a way to account for these individual situations. While smart pumps may be working appropriately to raise awareness of a dose being given outside of the usual range, repeated alerts are a new source of alert fatigue at the patient level; this may decrease nurse sensitivity to the alert, placing the patient at especially high risk for a nurse to miss a true error. Future work should focus on ways to mitigate this issue, identifying ways to individualize smart pump performance.

Given the opportunity, the vast majority of nurses override alerts, calling into question the ability to appropriately recognize alerts that require attention. This parallels the observed phenomenon seen with medication alerts produced during the prescribing phase of the medication lifecycle [26, 27]. Of concern, many high-risk medications that have been associated with harmful errors are generating a high number of smart pump alerts. The medications responsible for a high alert burden have high individual alert rates per infusion starts, suggesting that their dosing rules may not be optimized. Additionally, soft minimum alerts, which have high override rates [22] and low potential to reduce harm, contributed significantly to the alert burden in our study. Ideally, the best alerts identify true errors and require action.

In looking at the amount that programmed doses exceed limits, we found that several medications that generated high alert rates, such as morphine, alerted for doses that were just 20% above the soft dosing limit. This may indicate that adjustment of the dosing limits in the drug library could significantly decrease alert burden while continuing to promote patient safety by alerting for more significant overdoses. Several authors have demonstrated the utility of small dosing limit adjustments to decrease the number of nonactionable alerts, but more evidence is needed to understand the effect of changing dosing limits on patient safety [13, 28, 29].

Others have shown that alert number differs by time of day, day of the week, and month [15]. We found that overall alert number is highest on the evening shift but we contribute this to the fact that the majority of new fluids, TPN and lipids are hung during this shift. The overall percentage of alerts per infusion starts was not different based on shift or day of the week. Our data also suggest that nurses may experience some desensitization to infusion pump alerts over time. In desensitization, repeated exposure to alerts leads to declining responsiveness over time [14, 30–32]. We found a small but statistically significant increase in the number of alerts that were overridden and a decrease in the number of alerts that were reprogrammed or cancelled over time, suggesting that nurses may experience some densitization with continued infusion pump experience. No significant changes to the pump library limits or to hospital policy occurred during that time to otherwise account for the change. Given the small rate of change, further evaluation over time is required to determine if this is a true phenomenon.

### Strengths/limitations

Our study has a number of strengths. We took advantage of wirelessly downloaded smart pump information to build a large neonatal database evaluating over 370,000 infusions over multiple years. The granularity of this large dataset allowed us to identify unique trends in the data to understand some of the benefits and inherent risks of smart pumps. The current literature on medication administration errors in neonates is based primarily on small observational studies. This study is the first neonatal study to use non-observational methodology and a large dataset to assess the effect of smart pumps on neonatal safety.

There are limits to this approach as well. First, by utilizing a data approach, we lack the ability to assess human factors and contextual factors, such as patient and unit acuity, or workload complexity, which may influence the caregiver response to alerts and can affect how data is interpreted. While we met with frontline smart pump users to understand data trends and workarounds, we did not perform intensive observations or structured interviews. Second, there is not a one-to-one correlation between orders and infusions, which makes it impossible to ascertain the number of expected infusions to confirm complete data capture. Third, our study was limited to a single intensive care unit in a single site, which may limit generalizability to other environments and populations. Finally, we chose to focus on continuous medications, and the issues around dosing limits, alerts and alert fatigue may differ for intermittent medications compared to continuous medications.

Given the large number of infusions utilized in neonates, and essentially in all hospitalized patients, it is clear that measures that improve medication and fluid safety can have a significant impact. What appears to be a small number of prevented errors becomes large when analyzed at the level of the institution or hospital system. To further optimize the effectiveness of smart pumps, future work should evaluate the effect of smart pump alert burden on an individual caregiver level as individual alert fatigue or desensitization may contribute to medication errors.

## Conclusions

Smart pumps, when used appropriately, have the ability to improve neonatal medication safety in a small but important way. However, this benefit must be balanced with the effects of alert burden and alert fatigue, as individual medications or individual patients with a high alert burden may limit safety benefit through provider desensitization to alerts. Future work should address ways to improve alert salience, especially when alert rates are high, and identify ways to target smart pump performance when individualization is required, particularly to decrease repeated nonactionable alerts. Regular assessment of smart pump data to assess violations of limits, current library limits, alert frequency, provider actions, and use of basic infusions will help improve the usefulness of smart pump technology and the ability to prevent medication administration errors in our most vulnerable patients.

## Data Availability

The datasets generated during and/or analyzed during the current study are available from the corresponding author on reasonable request.

## Abbreviations

DERS: Dose error reduction software
ICU: Intensive care unit
NICU: Neonatal intensive care unit
